# The Planetary Health Diet and Its Association with Asthma and Airway Inflammation in School-Aged Children

**DOI:** 10.3390/nu16142241

**Published:** 2024-07-12

**Authors:** Mónica Rodrigues, Patrícia Padrão, Francisca de Castro Mendes, André Moreira, Pedro Moreira

**Affiliations:** 1 Epidemiology Research Unit, Institute of Public Health, University of Porto, 4050-600 Porto, Portugal; carolina101997@hotmail.com (M.R.); patriciapadrao@fcna.up.pt (P.P.); francisca_castromendes@hotmail.com (F.d.C.M.); andremoreira@med.up.pt (A.M.); 2 Laboratory for Integrative and Translational Research in Population Health, Institute of Public Health, University of Porto, 4050-600 Porto, Portugal; 3 Faculty of Nutrition and Food Sciences, University of Porto, 4150-180 Porto, Portugal; 4 Basic and Clinical Immunology, Department of Pathology, Faculty of Medicine, University of Porto, 4200-319 Porto, Portugal; 5 Immuno-Allergology Department, São João Hospital Center, 4200-319 Porto, Portugal

**Keywords:** dietary patterns, asthma, airway inflammation, obesity, pediatrics, sustainability

## Abstract

Poor dietary choices have been rising concurrently with an increase in asthma prevalence, especially in children. Dietary indexes that simultaneously measure the healthiness and sustainability of dietary patterns have emerged to address the dual concerns of human and planetary health. Accordingly, we aimed to evaluate adherence to a sustainable dietary pattern and its impact on airway inflammation and asthma. In this study, 660 school-aged children (49.1% females, 7–12 years) were considered. A cross-sectional analysis was performed to assess the association between diet and asthma and airway inflammation according to overweight/obesity. Diet was evaluated through the Planetary Health Diet Index (PHDI). Higher scores represent a healthier and more sustainable diet. Three definitions of asthma were considered based on a self-reported medical diagnosis, symptoms, asthma medication, measured lung function, and airway reversibility. Airway inflammation was assessed by exhaled fractional nitric oxide (eNO). We considered two categories of body mass index: non-overweight/non-obese and overweight/obese. The associations between diet with asthma and airway inflammation were estimated using adjusted binary logistic regressions. The odds of having airway inflammation decreased with the increase in PHDI score. Moreover, children in the non-overweight/non-obesity group in the fourth quartile of the PHDI had lower odds of having airway inflammation compared to children in the first quartile. Our study indicates that a healthier and sustainable diet is associated with lower levels of eNO, but only among children without overweight/obesity.

## 1. Introduction

Sustainable diets aim to tackle the growing concern surrounding both health and environmental aspects of food consumption and production. While various potential sustainable diets have been proposed, there is a crucial need to consider both environmental and health factors when recommending these diets [1]. 

Dietary patterns that are characterized by low consumption of fruits and vegetables, nuts, legumes, and whole grains, while featuring a high intake of red and processed meats, are the primary contributors to the significant health challenges faced globally [2]. 

Food security, climate stability, ecosystem well-being, and the overall health and nutrition of populations have been disrupted due to a shift in food patterns, particularly a rise in meat consumption [3,4]. Dietary choices are one of the major contributors to the human impact on greenhouse gas emissions and, consequently, climate change [3]. 

Regarding human health, an unbalanced diet has been related to diverse poor health outcomes, demonstrating the exertion of a negative influence on respiratory outcomes, such as airway inflammation and asthma [5], as well as on body weight [6], obesity being one of the principal factors leading to worst respiratory symptoms [5,7]. People who are obese face a heightened risk of asthma, along with more frequent and severe symptoms and exacerbations. Additionally, obese asthma is associated with a lower quality of life and diminished responsiveness to asthma medications [8,9]. Asthma prevalence in children has increased in many countries [10]. In parallel, the prevalence of pediatric obesity has also globally augmented over the past five decades [11].

Most of the research investigating the relationship between diet and asthma tends to focus on individual foods or components within foods [12]. Nonetheless, food patterns have been getting more attention for their overall potential effects on health [13] and on planet sustainability [14]. 

The Mediterranean diet, which is simultaneously a healthy and sustainable dietary pattern, has proved to be protective of airway inflammation [15] and asthma disease [16].

Nonetheless, adherence to the Mediterranean diet is decaying [17], and other dietary patterns, primarily established focusing on planetary health and sustainability, have been developed [18]. Taking into consideration the World Health Organization (WHO) and the Food and Agriculture Organization (FAO) contribution, healthy sustainable diets are “(…) dietary patterns that promote all dimensions of individuals’ health and wellbeing; have low environmental pressure and impact; are accessible, affordable, safe and equitable; and are culturally acceptable [19] ”. The EAT–Lancet Commission, in its report on “Healthy Diets from Sustainable Food Systems”, introduced a diet model called the “Planetary Health Diet”. This diet is designed to promote both planetary well-being and the health of the population. Its recommendations emphasize a higher intake of greens, vegetables, whole grains, and fruits, while advocating for reduced consumption of tubers, fish, meat, refined cereals, and eggs [20]. Cacau et al. proposed, then, the Planetary Health Diet Index (PHDI) based on this projected reference diet [18]. The PHDI was associated with increased dietary quality and with decreased greenhouse gas emissions [18]. Nonetheless, this index has yet to be tested regarding its association with health outcomes [21].

The impact of food on asthma and airway inflammation is of growing interest [9]. Introducing a new dimension, such as the consideration of diet sustainability, is a pressing requirement as well as a priority step towards fostering a healthier and more sustainable planet.

In this context, this study aimed to investigate adherence to the Planetary Health Diet, using the Planetary Health Diet Index (PHDI), and its relation to asthma and airway inflammation in children. Additionally, we explored the association between the PHDI, airway inflammation, and three different definitions of asthma, considering a stratification according to body mass index (BMI). We hypothesize that children with higher values for the PHDI score will have lower odds of having airway inflammation and asthma. 

## 2. Materials and Methods

### 2.1. Participant Assessment

The data included in this cross-sectional study were collected from January 2014 to March 2015. A total of 1602 school-aged children from 20 public schools, from Porto in Portugal, in the 3rd and 4th grades, aged 7–12 years old, were invited to participate [22]. A total of 686 (42.8%) did not have the signed informed consent, and 58 (3.8%) refused to perform clinical procedures. Among the remaining 858 individuals (53.6%), 660 (76.9%) had complete nutritional data and were considered for the analysis regarding adherence to the PHDI. Considering the adjusted associations between PHDI and our respiratory health outcomes (asthma and airway inflammation), 454 children were contemplated in the analysis. Written consent was obtained from every child’s legal guardian. The Ethics Committee of the University Hospital São João approved the study (ARIA 248-13), and all procedures were carried out under the Helsinki Declaration. 

#### 2.1.1. Dietary Intake and Diet Sustainability and Quality Assessment

Participants reported their food and beverage consumption over the past day to a trained interviewer through a 24 h food recall questionnaire. Children answered questions in accordance with standard protocols and provided thorough information, including specific brands and quantities; to support this task, a photographic guide was utilized to gauge portion sizes [23]. Following data collection, nutritional information and total energy intake (kcal) were calculated using the Food Processor^®^ software from ESHA Research (the version number of the software is V3), USA, which includes databases containing nutritional data for Portuguese food compositions. As information was registered in grams, to transform grams into kilocalories, the Portuguese Food Composition Table was used [24]. We validated the 24 h food recall questionnaires, employing Goldberg criteria to detect and exclude “under-reports” and “over-reports” of the reported diet [25].

Diet sustainability and quality were evaluated by The Planetary Health Diet Index (PHDI) [18], which is derived from the dietary guidelines of the EAT–Lancet Commission [20]. This index has 16 components that can receive a maximum score of either 10 or 5 points, leading to a total score that falls within the range of 0 to 150 points. The PHDI is subdivided into optimum, adequacy, ratio, and moderation components. The optimum components are food groups where it is preferable to have a minimum level of intake rather than none at all. Nonetheless, exceeding certain upper consumption limits, as recommended in the reference diet, could gradually harm sustainability and diet quality. These components encompass eggs, fish and seafood, tubers and potatoes, dairy, and unsaturated oils. The adequacy components are food groups where having no consumption is considered less favorable for dietary quality, but consuming them at or above a certain reference level is unlikely to have significant negative impacts on human and planetary health. These components include nuts and peanuts, legumes, fruits, total vegetables, and whole grains. The moderation components include red meat, chicken and substitutes, animal fats, and added sugars. In contrast to the adequacy components, the moderation components in the PHDI were chosen based on the idea that diets with little-to-no consumption of these food groups are associated with higher dietary quality and sustainability. The two ratio components within the PHDI reflect how dark green vegetables and red and orange vegetables are distributed in relation to the overall vegetable intake [18]. PHDI is calorie-based; the midpoints and ranges for the food group are calculated as their energetic contribution to the reference diet, which allows us to assess the adherence to the EAT–Lancet dietary pattern irrespective of the number of calories [18]. More information regarding the composition and calculation of the PHDI is available elsewhere [18]. 

We then subdivided the PHDI into quartiles. First quartile ≤25.76; second quartile >25.76 and ≤31.81; third quartile >31.81 and ≤41.21; and fourth quartile >41.21.

#### 2.1.2. Anthropometry

BMI was obtained by the calculation of weight/height^2^-kilograms per square meter (kg/m^2^). Weight was measured by a digital scale (Tanita™ BC-418 Segmental Body Analyzer, Middlesex, UK) in kilograms, and height was measured by a portable stadiometer and recorded in centimeters (cm). Participants were then divided into two groups, non-overweight/non-obese (*p* < 85th) and overweight/obese (*p* ≥ 85th) [26]; this was carried out according to specific age and sex percentiles provided by the US Centers for Disease Control and Prevention [27]. The US CDC definition was chosen based on an evaluation of the degree of agreement among several BMI classifications (US CDC, International Obesity Task Force, World Health Organization, and Percentage of Body Fat), with the US CDC showing the highest level of agreement with all the other classifications [28]. 

#### 2.1.3. Airway Inflammation

To quantify airway inflammation, FeNO was measured using a NObreath analyzer (Bedfont Scientific Ltd., Rochester, Kent, UK). The results were stratified following the official American Thoracic Society (ATS) criteria for children [29] and expressed as parts per billion (ppb). Exhaled NO was dichotomized using a cut-off point of equal to or above 35 ppb representing increased levels of eNO. Asthma is associated with increased eNO levels, and eNO numbers of over 35 ppb in children are considered high and indicate airway inflammation [29].

#### 2.1.4. Current Asthma and Respiratory Symptom Assessment

The ISAAC-based questionnaire was given to the child’s legal guardian. It enquired about social, demographic, and behavioral information and consisted of questions about allergic/respiratory health and respiratory symptoms [30]. 

Airway reversibility and lung function were recorded before and after 15 min of the inhalation of 400 μg of salbutamol and evaluated through spirometry, following the official ATS/European Respiratory Society (ERS) guidelines [31]. A positive bronchodilation (+BD) test was considered when at least a 12% and over 200 mL increase in forced expiratory volume in one second (FEV1s) was verified. 

Three different definitions of asthma were considered as previously described [32]: (i) ever asthma: self-reported medical diagnosis; (ii) medical diagnosis with asthma symptoms or +BD test: self-reported medical diagnosis with reported symptoms (wheezing, dyspnea, or dry cough) occurring in the past 12 months or positive BD test; and (iii) medical diagnosis and using medication for asthma—self-reported medical diagnosis and currently under anti-asthma medication.

#### 2.1.5. Atopy

Skin-prick tests (SPTs) were performed to define atopy (a positive SPT-wheal of at least 3 mm diameter for at least one allergen). The test was carried out on children’s forearms using a QuickTest^TM^ containing a house dust mite, a mix of weeds, a mix of grasses, *Alternaria alternata*, cat and dog dander, a negative control, and a positive control containing histamine at 10 mg/mL (Hall Allergy^®^, Leiden, The Netherlands), and the results were read after 15 min [33]. 

#### 2.1.6. Physical Activity

The evaluation of physical activity was led through a questionnaire answered by the parents or legal guardians [34]. The practice of sports activities besides the ones performed in the physical education classes was evaluated. This information was used to characterize children’s level of physical activity into three groups: <2 times/week, 2–3 times/week, or ≥4 times/week [34]. 

#### 2.1.7. Socio-Economic Data

Parental education level was described as the number of completed school years. It was then divided into 3 categories established by the parent with the highest education level: ≤9 years, between ≥10 and ≤12 years, and >12 years, and was used to denote the socio-economic status. 

#### 2.1.8. Statistical Analyses

The statistical analyses were performed using the statistical software SPSS ^®^ v27.0. 

To check normality for continuous variables, the skewness and kurtosis test was used. The characteristics of the participants are presented for the whole sample considering the PHDI categories as the number and percentages for categorical variables, as mean ± standard deviation (SD) for normal distributed continuous variables and as median (25th–75th percentile) for non-Gaussian distributed continuous variables. 

The associations between PHDI score (our independent variable) and airway inflammation and the three asthma definitions (dependent variables) were estimated using adjusted logistic regression models (OR, 95% CI). Confounders were considered such as age, sex, parental education, BMI, atopy, physical activity, and nutritional-supplementation use [35]. Significant differences were defined with an α-value of less than 5%, with a 95% confidence interval (*p* < 0.05).

## 3. Results

In our sample, children had a mean ± SD PHDI score of 32.72 ± 11.45, and the maximum obtained score was 65.59. Regarding children with obesity/overweight, the mean ± SD score was 31.75 ± 11.85; for children without overweight/obesity, the value was 33.05 ± 11.30 (Table 1). 

In our sample, there were not any statistically significant differences between children regarding the PHDI groups, except for age; and regarding parental education, children in the higher quartile of the PHDI score had parents with higher education level (*p* < 0.001) (Table 2).

The odds of having airway inflammation decreased with the increase in PHDI score (OR = 0.97, 95%CI: 0.93–0.99, *p*-trend = 0.047), in the non-overweight/non-obese group. Additionally, children with non-overweight/non-obesity in the highest quartile of the PHDI had decreased odds of having eNO ≥ 35 ppb (OR = 0.36, 95%CI: 0.13–0.98, *p* = 0.045) compared to children in the first quartile (Table 3).

## 4. Discussion

In our sample of school-aged children, we found that a more healthy and sustainable diet, as classified by the PHDI, is associated with lower levels of airway inflammation among the non-overweight/non-obese group of school-aged children.

Sustainable diets are usually rich in vegetables, legumes, greens, fruits, and whole grains [4]. These are foods and food groups characterized by their richness in micronutrients, bioactive compounds, and fiber, contributing to antioxidant and anti-inflammatory activities, which have been related to positive outcomes regarding asthma and airway inflammation [36]. In fact, the Mediterranean diet, an example of a well-known sustainable and healthy dietary pattern, has also demonstrated positive effects on airway inflammation and asthma [15,37,38,39,40,41]. In children, a systematic review with a meta-analysis of observational studies verified that higher adherence to the Mediterranean diet during childhood is a protective factor for asthma [38]. One other systematic review found similar results in children and adolescents [16]. Furthermore, adherence to the Mediterranean diet has been demonstrated to modulate the production of some inflammatory mediators known to have a pathogenetic role in the airways. Higher adherence to this diet pattern was associated with lower IL-4 and IL-17 in asthmatic children [41]. On the other side, school-aged children with a Western diet, which is a pattern commonly characterized by its increased intake of processed and red meats, and saturated fats, components connected with an unsustainable dietary pattern, seem to be at a higher risk of having asthma [42]. 

It is important to consider that we only found a protective effect of the Planetary Health Diet on the non-overweight/non-obesity group. We may assume that in the case of children with obesity, parents may change their diet to a healthier one, resulting in reverse causation: children who, throughout the years, have followed a more Westernized, obesogenic, and unhealthier diet may have recently changed their diet to a healthier and more sustainable one. Hence, we would not be able to see a protective effect of diet on airway inflammation and asthma. Additionally, individuals with obesity may tend to report a more healthy diet than they, in truth, have [43]. It has been demonstrated that under-reporting of energy intake and diet components happens especially in individuals with obesity [43]. Nonetheless, we used the Goldberg method to identify under-reports and over-reports in the 24 h dietary recall questionnaire [25]. 

Moreover, another possible explanation for this lack of association is that most of the investigations reporting on the association between eNO levels and obesity found no positive association [44,45]. On asthmatic children with obesity, it was verified that there was no correlation and even a negative correlation between exhaled NO and obesity, and similar results were found in adults [44,45]. A mechanical effect of excessive weight at the thorax level, a consequence of overweight/obesity, may inhibit the diffusion and even the production of nitric oxide [46]. One other study has suggested that in obesity, the typical low-grade inflammation and oxidative stress might result in an increased production of reactive oxygen species with significant conversion of airway nitric oxide into reactive nitrogen species [44]. Nonetheless, we cannot rule out the possibility of simply not finding any results in the overweight/obesity group because of a small sample size. Nevertheless, a study in adults also discovered that the association between some healthy dietary patterns and asthma outcomes changed when performing the analysis stratifying individuals based on BMI. Most of the statistically significant associations demonstrating a protective effect of diet were lost when BMI was ≥25 kg/m^2^ [5].

It is also valuable to note that the children from our study had, overall, quite a low mean score on the PHDI. Nonetheless, similar low results have been observed in previous studies using the PHDI [47,48,49]. These results may represent the difficulty in achieving a diet that is both healthy and sustainable. The previous observation led us to consider which factors might explain the lack of an association between the PHDI score and asthma. The Planetary Health Diet Index limits the intake of some foods and food groups, as is the case of fish and unsaturated oils. Both fish [50,51,52,53,54,55] and olive oil (an unsaturated oil) [56] have demonstrated positive effects on asthma and asthma-related outcomes. A meta-analysis demonstrated that fish consumption in infancy was related to a lower occurrence of asthma during childhood [52]. Another meta-analysis found that “all fish” (lean and fatty) demonstrated a protective effect on asthma in children, [55] and regarding children and adolescents, fatty fish (rich in omega 3) intake was found to be more protective than no fish consumption [55]. As for olive oil, a multi-case study in adults demonstrated that higher consumption of olive oil and oleic acid was associated with a reduced risk of current asthma [56]. This index does not limit these foods because of their nutritional constituents or because they are not healthy, but rather because a high consumption may not be sustainable for the planet [18]. Taking this into consideration, not all children with an asthma-protective diet will have a sustainable diet. Children with a high consumption of fish and olive oil may fall into the lower categories of the PHDI score. 

We acknowledge some limitations of our study. Primarily, as we previously noted, this is a cross-sectional study and we are not able to infer causality regarding the protective effect of the Planetary Health Diet on airway inflammation. It is also plausible to assume that reverse causation may occur [57]; we might hypothesize that some children formerly diagnosed with asthma and obesity changed their dietary pattern to a healthier one, affecting the results. However, epidemiologic studies are necessary for establishing possible causes of diseases, specifically when experimental study designs are challenging [58]. Additionally, to minimize bias in their outcome evaluation, the same research team collected detailed health data at the same time period (demographics, diet, respiratory outcomes, and anthropometrics). 

One of our main limitations lies in the application of a single time-point assessment to capture dietary intake [59]. A single day does not characterize typical intake, and multiple recalls would be more adequate to detail an individual’s dietary habits [60]. Moreover, we utilized a single 24 h food recall questionnaire, a method predominantly focused on short-term consumption, which is not able to capture seasonality differences. This is a more practical and easy approach, but it fails to account for the dynamism of dietary patterns [59]. Intake evolves throughout the stages of life, especially from childhood to adolescence [61], and the food environment may also undergo constant modifications, changing the diet [59,61]. To achieve a more accurate representation of dietary patterns, researchers are progressively emphasizing the need for repeated dietary assessments throughout life [59]. A longitudinal approach, as a cohort study design, with multiple assessments over time, would offer a more holistic picture of how dietary patterns may differently influence respiratory outcomes [59]. Nonetheless, a positive point of using a 24 h recall questionnaire is that it is able to estimate the current dietary habits without tempting children to modify eating behaviors due to the lengthy process of keeping records or the awareness that their diet is being evaluated [62]. Additionally, information about common container sizes, ingredients used in mixed dishes, and commercial product brand names was collected, allowing for a high-quality characterization of intake, but we also acknowledge a possible limitation in estimating vegetable oils, as most of the reports seem to focus only on those added after cooking (seasoning). So, there may be an under-reporting of this category in our sample. 

Another important limitation of this study is the possibility of recall bias. Children’s reports may be more prone to inaccuracies owing to restricted memory and food knowledge [63]. However, recognizing the difficulty in accurately estimating portion sizes and aiming to prevent misreporting, nutritionists and specially trained interviewers conducted the 24 h food recall questionnaires with the children. They used food photograph models to quantify portion sizes, using their experience in drawing information without leading the children to answer specific responses [64]. Our evaluation of physical activity, as it is based on a questionnaire, may also be prone to recall bias. However, since sports and physical activities often include fixed training sessions with set schedules, they may be easier to remember and report.

Another possible limitation is that BMI was used to categorize children regarding their overweight/obesity status, and BMI does not consider body composition [65,66,67,68]. Nonetheless, objective measures of height and weight were obtained, avoiding parental self-perceptions, as parents tend to misjudge their children’s overweight/obesity category [69].

Finally, although confounders were chosen based on prior research and understanding of their association with diet and the outcomes studied, residual confounding might still exist. 

Our study also showcases multiple strengths. Firstly, to the best of our knowledge, this is the first-ever report evaluating the impact of the Planetary Health Diet Index on asthma and airway inflammation. Moreover, our research also has the benefit of taking into consideration significant potential confounders, including parental education level, atopy, nutritional supplementation, physical activity level, breastfeeding, overweight/obesity status, and mother smoking during pregnancy, all of which are deemed relevant when addressing asthma and asthma-related outcomes [35,70].

Furthermore, in agreement with Silva et al. [32], a standardized definition of asthma must include airway reversibility and a questionnaire, and our investigation took into account the ISAAC-based questionnaire and the objectively measured spirometry with bronchodilation, combining it to characterize different asthma definitions [32]. 

Finally, dietary patterns are becoming increasingly recognized as a crucial method to understand dietary habits and their relation to health [71]. The difficulties faced nowadays regarding sustainability add another layer to this subject of study [1]. Unlike traditional approaches that focus on individual nutrients or foods, dietary patterns offer a comprehensive view by considering the complex interactions and synergies within the entire food matrix [72]. Using the Planetary Health Diet Index to characterize diet allowed us to also take into account diet sustainability [18]. This diet index acknowledges that foods are consumed in combinations and that their effects on health outcomes may be influenced by how they interact together and how, overall, they may impact health and disease risk [71], as well as planetary health [18,62]. Thus, dietary pattern analysis not only improves our ability to characterize diet effects more accurately but also provides insights into the broader implications for nutrition and public health policies, and, in this specific case, environmental health [1,71]. 

## 5. Conclusions

In conclusion, this study demonstrates a protective effect of a sustainable and healthy diet on airway inflammation, in non-overweight/non-obese school-aged children. Nonetheless, the same results are not observed for asthma, underlining the crucial need to consider both health and environmental factors when recommending a dietary pattern to specific individuals. This research highlights the importance of promoting a diet that is rich in vegetables and fruits, whole grains, greens and beans, and healthy fats, while restricting saturated fats, and red and processed meats. 

Recognizing the impact of food consumption on asthma and airway inflammation, alongside its environmental effects, could aid in developing more precise clinical and public health guidelines regarding dietary patterns for better health. However, it is important to note that significant gaps remain regarding which specific foods or diets individuals should adopt to improve their respiratory health.

## Figures and Tables

**Table 1:** Mean PHDI score by overweight/obesity status.

PHDI Score	Total Participants	Non-Overweight/Non-Obesity	Overweight/Obesity
PHDI score, mean ± SD	32.72 ± 11.45	33.05 ± 11.30	31.74 ± 11.85

**Table 2:** Summary of participants’ characteristics by quartiles of PHDI.

Characteristics	Total	Planetary Q1	Planetary Q2	Planetary Q3	Planetary Q4	*p*-Value
Age, years	8.68 ± 0.77	8.82 ± 0.83	8.71 ± 0.79	8.67 ± 0.77	8.53 ± 0.67	0.009
Sex, girls, n (%)	324 (49.1%)	81 (50.3%)	83 (43.6%)	72 (53.3%)	88 (13.3%)	0.355
Parental education, n (%)						0.001
<9 years	188 (35.5%)	50 (37.9%)	51 (39.5%)	45 (34.9%)	42 (30.2%)	
10–12 years	161 (30.4%)	52 (39.4%)	42 (32.6%)	36 (27.9%)	31 (22.3%)	
>12 years	180 (34.0%)	30 (22.7%)	36 (27.9%)	48 (37.2%)	66 (47.5%)	
BMI, n (%)						0.715
Non-overweight/non-obese	491 (74.4%)	119 (72.1%)	122 (73.9%)	122 (73.9%)	128 (77.6%)	
Overweight/obese	169 (25.6%)	46 (27.9%)	43 (26.1%)	43 (26.1%)	37 (22.4%)	
Nutritional Supplementation, n (%)	87 (14.9%)	21 (14.7%)	19 (13.2%)	24 (16.4%)	23 (15.1%)	0.893
Breastfeeding, n (%)	474 (81.9%)	116 (81.7%)	118 (81.4%)	116 (81.7%)	124 (82.7%)	0.993
Smoking during pregnancy, n (%)	157 (25.6%)	42 (27.8%)	35 (22.9%)	46 (30.3%)	34 (21.7%)	0.261
Physical Activity						0.169
<2 times/week	294 (49.7%)	82 (55.0%)	74 (51.7%)	78 (52.7%)	60 (39.5%)	
2–3 times/week	221 (37.3%)	49 (32.9%)	50 (35.0%)	53 (35.8%)	69 (45.4%)	
≥4 times/week	77 (13.0%)	18 (12.1%)	19 (13.3%)	17 (11.5%)	23 (25.7%)	
Atopy, n (%)	227 (34.9%)	50 (30.7%)	63 (38.4%)	62 (37.6%)	52 (32.7%)	
Airway Inflammation, FeNO ≥ 35 ppb, n (%)	86 (13%)	24 (14.5%)	19 (11.5%)	26 (15.8%)	17 (10.3%)	0.418
Asthma Definitions						
Ever	45 (7.1%)	10 (6.3%)	8 (5.0%)	17 (10.8%)	10 (6.2%)	0.189
Medical diagnosis with asthma symptoms or +BD	56 (8.5%)	12 (7.3%)	12 (7.3%)	17 (10.3%)	15 (9.1%)	0.704
Medical diagnosis and under asthma treatment	37 (5.6%)	7 (4.2%)	7 (4.2%)	15 (9.1%)	8 (4.8%)	0.163

Note: FeNO, fractional exhaled nitric oxide; +BD, positive bronchodilation test.

**Table 3:** Analysis of the association between the PHDI with asthma and airway inflammation.

Respiratory Outcomes	Non-Overweight/ Non-Obese	*p*-Value	Overweight/ Obese	*p*-Value	All	*p*-Value
Airway Inflammation, FeNO ≥ 35 ppb						
Planetary Q1			Reference			
Planetary Q2	0.43 (0.16–1.15)	0.091	0.68 (0.06–7.41)	0.750	0.61 (0.26–1.44)	0.262
Planetary Q3	0.46 (0.18–1.17)	0.104	3.70 (0.30–45.24)	0.305	0.74 (0.33–1.63)	0.453
Planetary Q4	0.36 (0.13–0.98)	0.045	1.07 (0.09–12.66)	0.959	0.53 (0.22–1.27)	0.156
*p-trend*	0.97 (0.93–0.999)	0.047	1.01 (0.94–1.09)	0.729	0.98 (0.95–1.01)	0.184
Asthma Definitions:						
Ever						
Planetary Q1			Reference			
Planetary Q2	1.45 (0.30–7.06)	0.648	1.20 (0.14–10.29)	0.868	1.25 (0.36–4.29)	0.728
Planetary Q3	1.85 (0.43–8.00)	0.409	4.89 (0.72–33.18)	0.104	2.31 (0.77–6.91)	0.133
Planetary Q4	1.47 (0.33–6.63)	0.618	2.02 (0.27–15.36)	0.496	1.52 (0.47–4.96)	0.484
*p-trend*	1.01 (0.97–1.06)	0.592	1.03 (0.97–1.10)	0.302	1.02 (0.98–1.06)	0.301
Medical diagnosis with asthma symptoms or +BD						
Planetary Q1			Reference			
Planetary Q2	0.96 (0.23–3.55)	0.948	0.69 (0.12–3.99)	0.674	0.82 (0.30–2.26)	0.702
Planetary Q3	1.34 (0.41–4.40)	0.630	0.82 (0.13–5.23)	0.837	1.08 (0.42–2.78)	0.867
Planetary Q4	0.80 (0.23–2.81)	0.722	2.53 (0.38–17.06)	0.828	0.84 (0.32–2.25)	0.734
*p-trend*	0.99 (0.97–1.04)	0.809	1.21 (0.21–1.68)	0.790	1.00 (0.97–1.03)	0.899
Medical diagnosis and under asthma treatment						
Planetary Q1			Reference			
Planetary Q2	1.44 (0.23–9.16)	0.701	1.06 (0.12–9.11)	0.957	1.20 (0.31–4.70)	0.793
Planetary Q3	2.51 (0.48–13.2)	0.277	2.36 (0.32–17.35)	0.399	2.28 (0.68–7.63)	0.183
Planetary Q4	1.06 (0.17–6.83)	0.951	1.61 (0.20–12.82)	0.654	1.18 (0.31–4.48)	0.809
*p-trend*	1.01 (0.95–1.06)	0.862	1.02 (0.96–1.09)	0.529	1.01 (0.97–1.05)	0.580

Notes: Association between quartiles of the Planetary Health Diet Index and airway inflammation and asthma, stratified by BMI categories adjusted for sex, age, supplementation use, parental education, physical activity, mother smoking during pregnancy, breastfeeding, and atopy (454 children were included in the cross-sectional analysis). When considering the data not stratified by BMI, the logistic regression was further adjusted for BMI.

## Data Availability

The data that support the findings of this study will be made available by the authors upon reasonable request.

## References

[1] Springmann M., Wiebe K., Mason-D’Croz D., Sulser T.B., Rayner M., Scarborough P. (2018). Health and nutritional aspects of sustainable diet strategies and their association with environmental impacts: A global modelling analysis with country-level detail. Lancet Planet. Health.

[2] Forouzanfar M.H., Alexander L., Anderson H.R., Bachman V.F., Biryukov S., Brauer M., Burnett R., Casey D., Coates M.M., Cohen A. (2015). Global, regional, and national comparative risk assessment of 79 behavioural, environmental and occupational, and metabolic risks or clusters of risks in 188 countries, 1990–2013: A systematic analysis for the Global Burden of Disease Study 2013. Lancet.

[3] Garnett T. (2011). Where are the best opportunities for reducing greenhouse gas emissions in the food system (including the food chain)?. Food Policy.

[4] Teixeira B., Afonso C., Rodrigues S., Oliveira A. (2022). Healthy and Sustainable Dietary Patterns in Children and Adolescents: A Systematic Review. Adv. Nutr..

[5] Andrianasolo R.M., Kesse-Guyot E., Adjibade M., Hercberg S., Galan P., Varraso R. (2018). Associations between dietary scores with asthma symptoms and asthma control in adults. Eur. Respir. J..

[6] Martinez K.B., Leone V., Chang E.B. (2017). Western diets, gut dysbiosis, and metabolic diseases: Are they linked?. Gut Microbes.

[7] Barros R., Moreira P., Padrão P., Teixeira V.H., Carvalho P., Delgado L., Moreira A. (2017). Obesity increases the prevalence and the incidence of asthma and worsens asthma severity. Clin. Nutr..

[8] Peters U., Dixon A.E., Forno E. (2018). Obesity and asthma. J. Allergy Clin. Immunol..

[9] Guilleminault L., Williams E.J., Scott H.A., Berthon B.S., Jensen M., Wood L.G. (2017). Diet and Asthma: Is It Time to Adapt Our Message?. Nutrients.

[10] Asher M.I., Montefort S., Björkstén B., Lai C.K., Strachan D.P., Weiland S.K., Williams H. (2006). Worldwide time trends in the prevalence of symptoms of asthma, allergic rhinoconjunctivitis, and eczema in childhood: ISAAC Phases One and Three repeat multicountry cross-sectional surveys. Lancet.

[11] Jebeile H., Kelly A.S., O’Malley G., Baur L.A. (2022). Obesity in children and adolescents: Epidemiology, causes, assessment, and management. Lancet Diabetes Endocrinol..

[12] Garcia-Larsen V., Del Giacco S.R., Moreira A., Bonini M., Charles D., Reeves T., Carlsen K.H., Haahtela T., Bonini S., Fonseca J. (2016). Asthma and dietary intake: An overview of systematic reviews. Allergy.

[13] Reyes-Angel J., Han Y.Y., Litonjua A.A., Celedón J.C. (2021). Diet and asthma: Is the sum more important than the parts?. J. Allergy Clin. Immunol..

[14] Fresán U., Sabaté J. (2019). Vegetarian Diets: Planetary Health and Its Alignment with Human Health. Adv. Nutr..

[15] Rodrigues M., de Castro Mendes F., Padrão P., Delgado L., Paciência I., Barros R., Rufo J.C., Silva D., Moreira A., Moreira P. (2023). Mediterranean Diet and Airway Inflammation in School-Aged Children. Children.

[16] Koumpagioti D., Boutopoulou B., Moriki D., Priftis K.N., Douros K. (2022). Does Adherence to the Mediterranean Diet Have a Protective Effect against Asthma and Allergies in Children? A Systematic Review. Nutrients.

[17] Vilarnau C., Stracker D.M., Funtikov A., da Silva R., Estruch R., Bach-Faig A. (2019). Worldwide adherence to Mediterranean Diet between 1960 and 2011. Eur. J. Clin. Nutr..

[18] Cacau L.T., De Carli E., de Carvalho A.M., Lotufo P.A., Moreno L.A., Bensenor I.M., Marchioni D.M. (2021). Development and Validation of an Index Based on EAT-Lancet Recommendations: The Planetary Health Diet Index. Nutrients.

[19] Food and Agricultural Organisation of the United Nations (FAO), World Health Organization (WHO) (2019). Sustainable Healthy Diets—Guiding Principles.

[20] Willett W., Rockström J., Loken B., Springmann M., Lang T., Vermeulen S., Garnett T., Tilman D., DeClerck F., Wood A. (2019). Food in the Anthropocene: The EAT-Lancet Commission on healthy diets from sustainable food systems. Lancet.

[21] Cacau L.T., Benseñor I.M., Goulart A.C., Cardoso L.O., Lotufo P.A., Moreno L.A., Marchioni D.M. (2021). Adherence to the Planetary Health Diet Index and Obesity Indicators in the Brazilian Longitudinal Study of Adult Health (ELSA-Brasil). Nutrients.

[22] Mendes F.C., Paciência I., Cavaleiro Rufo J., Farraia M., Silva D., Padrão P., Delgado L., Garcia-Larsen V., Moreira A., Moreira P. (2021). Higher diversity of vegetable consumption is associated with less airway inflammation and prevalence of asthma in school-aged children. Pediatr. Allergy Immunol..

[23] Walker J.L., Ardouin S., Burrows T. (2018). The validity of dietary assessment methods to accurately measure energy intake in children and adolescents who are overweight or obese: A systematic review. Eur. J. Clin. Nutr..

[24] Instituto Nacional de Saúde Dr. Ricardo Jorge (2022). Tabela da Composição de Alimentos.

[25] Black A.E. (2000). Critical evaluation of energy intake using the Goldberg cut-off for energy intake:basal metabolic rate. A practical guide to its calculation, use and limitations. Int. J. Obes. Relat. Metab. Disord..

[26] de Castro Mendes F., Paciência I., Rufo J.C., Silva D., Cunha P., Farraia M., Delgado L., Moreira P., Moreira A. (2019). Asthma and body mass definitions affect estimates of association: Evidence from a community-based cross-sectional survey. ERJ Open Res..

[27] Kuczmarski R.J., Ogden C.L., Grummer-Strawn L.M., Flegal K.M., Guo S.S., Wei R., Mei Z., Curtin L.R., Roche A.F., Johnson C.L. (2000). CDC Growth Charts: United States.

[28] de Castro Mendes F., Paciência I., Rufo J.C., Farraia M., Silva D., Padrão P., Delgado L., Garcia-Larsen V., Moreira A., Moreira P. (2021). Increasing Vegetable Diversity Consumption Impacts the Sympathetic Nervous System Activity in School-Aged Children. Nutrients.

[29] Dweik R.A., Boggs P.B., Erzurum S.C., Irvin C.G., Leigh M.W., Lundberg J.O., Olin A.C., Plummer A.L., Taylor D.R. (2011). An official ATS clinical practice guideline: Interpretation of exhaled nitric oxide levels (FENO) for clinical applications. Am. J. Respir. Crit. Care Med..

[30] Asher M.I., Keil U., Anderson H.R., Beasley R., Crane J., Martinez F., Mitchell E.A., Pearce N., Sibbald B., Stewart A.W. (1995). International Study of Asthma and Allergies in Childhood (ISAAC): Rationale and methods. Eur. Respir. J..

[31] Miller M.R., Hankinson J., Brusasco V., Burgos F., Casaburi R., Coates A., Crapo R., Enright P., van der Grinten C.P., Gustafsson P. (2005). Standardisation of spirometry. Eur. Respir. J..

[32] Silva D., Severo M., Paciência I., Rufo J., Martins C., Moreira P., Padrão P., Delgado L., Moreira A. (2019). Setting definitions of childhood asthma in epidemiologic studies. Pediatr. Allergy Immunol..

[33] Heinzerling L., Mari A., Bergmann K.C., Bresciani M., Burbach G., Darsow U., Durham S., Fokkens W., Gjomarkaj M., Haahtela T. (2013). The skin prick test—European standards. Clin. Transl. Allergy.

[34] Moreira P., Santos S., Padrão P., Cordeiro T., Bessa M., Valente H., Barros R., Teixeira V., Mitchell V., Lopes C. (2010). Food patterns according to sociodemographics, physical activity, sleeping and obesity in Portuguese children. Int. J. Env. Res. Public. Health.

[35] Nurmatov U., Nwaru B.I., Devereux G., Sheikh A. (2012). Confounding and effect modification in studies of diet and childhood asthma and allergies. Allergy.

[36] Rodrigues M., de Castro Mendes F., Delgado L., Padrão P., Paciência I., Barros R., Rufo J.C., Silva D., Moreira A., Moreira P. (2023). Diet and Asthma: A Narrative Review. Appl. Sci..

[37] Amazouz H., Roda C., Beydon N., Lezmi G., Bourgoin-Heck M., Just J., Momas I., Rancière F. (2021). Mediterranean diet and lung function, sensitization, and asthma at school age: The PARIS cohort. Pediatr. Allergy Immunol..

[38] Garcia-Marcos L., Castro-Rodriguez J.A., Weinmayr G., Panagiotakos D.B., Priftis K.N., Nagel G. (2013). Influence of Mediterranean diet on asthma in children: A systematic review and meta-analysis. Pediatr. Allergy Immunol..

[39] Papamichael M.M., Katsardis C., Lambert K., Tsoukalas D., Koutsilieris M., Erbas B., Itsiopoulos C. (2019). Efficacy of a Mediterranean diet supplemented with fatty fish in ameliorating inflammation in paediatric asthma: A randomised controlled trial. J. Hum. Nutr. Diet..

[40] Barros R., Moreira A., Fonseca J., de Oliveira J.F., Delgado L., Castel-Branco M.G., Haahtela T., Lopes C., Moreira P. (2008). Adherence to the Mediterranean diet and fresh fruit intake are associated with improved asthma control. Allergy.

[41] Douros K., Thanopoulou M.I., Boutopoulou B., Papadopoulou A., Papadimitriou A., Fretzayas A., Priftis K.N. (2019). Adherence to the Mediterranean diet and inflammatory markers in children with asthma. Allergol. Immunopathol..

[42] Patel S., Custovic A., Smith J.A., Simpson A., Kerry G., Murray C.S. (2014). Cross-sectional association of dietary patterns with asthma and atopic sensitization in childhood—In a cohort study. Pediatr. Allergy Immunol..

[43] Winkler J.T. (2005). The fundamental flaw in obesity research. Obes. Rev..

[44] Khalid F., Holguin F. (2018). A review of obesity and asthma across the life span. J. Asthma.

[45] Barros R., Moreira A., Fonseca J., Moreira P., Fernandes L., de Oliveira J.F., Delgado L., Castel-Branco M.G. (2006). Obesity and airway inflammation in asthma. J. Allergy Clin. Immunol..

[46] Maniscalco M., de Laurentiis G., Zedda A., Faraone S., Giardiello C., Cristiano S., Sofia M. (2007). Exhaled nitric oxide in severe obesity: Effect of weight loss. Respir. Physiol. Neurobiol..

[47] Marchioni D.M., Cacau L.T., De Carli E., Carvalho A.M., Rulli M.C. (2022). Low Adherence to the EAT-Lancet Sustainable Reference Diet in the Brazilian Population: Findings from the National Dietary Survey 2017–2018. Nutrients.

[48] Macit-Çelebi M.S., Bozkurt O., Kocaadam-Bozkurt B., Köksal E. (2023). Evaluation of sustainable and healthy eating behaviors and adherence to the planetary health diet index in Turkish adults: A cross-sectional study. Front. Nutr..

[49] Cacau L.T., Hanley-Cook G.T., Huybrechts I., De Henauw S., Kersting M., Gonzalez-Gross M., Gottrand F., Ferrari M., Nova E., Castillo M.J. (2023). Relative validity of the Planetary Health Diet Index by comparison with usual nutrient intakes, plasma food consumption biomarkers, and adherence to the Mediterranean diet among European adolescents: The HELENA study. Eur. J. Nutr..

[50] Venter C., Meyer R., Nwaru B., Roduit C., Untersmayr E., Adel-Patient K., Agache I., Agostoni C., Akdis C., Bischoff S. (2019). EAACI position paper: Influence of dietary fatty acids on asthma, food allergy, and atopic dermatitis. Allergy.

[51] Barros R., Moreira A., Fonseca J., Delgado L., Castel-Branco M.G., Haahtela T., Lopes C., Moreira P. (2011). Dietary intake of α-linolenic acid and low ratio of n-6:n-3 PUFA are associated with decreased exhaled NO and improved asthma control. Br. J. Nutr..

[52] Yang H., Xun P., He K. (2013). Fish and fish oil intake in relation to risk of asthma: A systematic review and meta-analysis. PLoS ONE.

[53] Kim J.L., Elfman L., Mi Y., Johansson M., Smedje G., Norbäck D. (2005). Current asthma and respiratory symptoms among pupils in relation to dietary factors and allergens in the school environment. Indoor Air.

[54] Tabak C., Wijga A.H., de Meer G., Janssen N.A., Brunekreef B., Smit H.A. (2006). Diet and asthma in Dutch school children (ISAAC-2). Thorax.

[55] Papamichael M.M., Shrestha S.K., Itsiopoulos C., Erbas B. (2018). The role of fish intake on asthma in children: A meta-analysis of observational studies. Pediatr. Allergy Immunol..

[56] Cazzoletti L., Zanolin M.E., Spelta F., Bono R., Chamitava L., Cerveri I., Garcia-Larsen V., Grosso A., Mattioli V., Pirina P. (2019). Dietary fats, olive oil and respiratory diseases in Italian adults: A population-based study. Clin. Exp. Allergy.

[57] Savitz D.A., Wellenius G.A. (2022). Can Cross-Sectional Studies Contribute to Causal Inference? It Depends. Am. J. Epidemiology.

[58] Matsui E.C., Keet C.A. (2017). Weighing the evidence: Bias and confounding in epidemiologic studies in allergy/immunology. J. Allergy Clin. Immunol..

[59] Chong M.F.-F. (2022). Dietary trajectories through the life course: Opportunities and challenges. Br. J. Nutr..

[60] Shim J.S., Oh K., Kim H.C. (2014). Dietary assessment methods in epidemiologic studies. Epidemiol. Health.

[61] Biazzi Leal D., Altenburg de Assis M.A., Hinnig P.F., Schmitt J., Soares Lobo A., Bellisle F., Di Pietro P.F., Vieira F.K., de Moura Araujo P.H., de Andrade D.F. (2017). Changes in Dietary Patterns from Childhood to Adolescence and Associated Body Adiposity Status. Nutrients.

[62] Ortega R.M., Pérez-Rodrigo C., López-Sobaler A.M. (2015). Dietary assessment methods: Dietary records. Nutr. Hosp..

[63] Foster E., Bradley J. (2018). Methodological considerations and future insights for 24-hour dietary recall assessment in children. Nutr. Res..

[64] Biró G., Hulshof K.F., Ovesen L., Amorim Cruz J.A. (2002). Selection of methodology to assess food intake. Eur. J. Clin. Nutr..

[65] Müller M.J., Lagerpusch M., Enderle J., Schautz B., Heller M., Bosy-Westphal A. (2012). Beyond the body mass index: Tracking body composition in the pathogenesis of obesity and the metabolic syndrome. Obes. Rev..

[66] Curry B.A., Blizzard C.L., Schmidt M.D., Walters E.H., Dwyer T., Venn A.J. (2011). Longitudinal associations of adiposity with adult lung function in the Childhood Determinants of Adult Health (CDAH) study. Obesity.

[67] Papoutsakis C., Priftis K.N., Drakouli M., Prifti S., Konstantaki E., Chondronikola M., Antonogeorgos G., Matziou V. (2013). Childhood Overweight/Obesity and Asthma: Is There a Link? A Systematic Review of Recent Epidemiologic Evidence. J. Acad. Nutr. Diet..

[68] Barros R., Delgado L. (2016). Visceral adipose tissue: A clue to the obesity-asthma endotype(s)?. Rev. Port. Pneumol..

[69] Lundahl A., Kidwell K.M., Nelson T.D. (2014). Parental underestimates of child weight: A meta-analysis. Pediatrics.

[70] Venter C., Greenhawt M., Meyer R.W., Agostoni C., Reese I., du Toit G., Feeney M., Maslin K., Nwaru B.I., Roduit C. (2020). EAACI position paper on diet diversity in pregnancy, infancy and childhood: Novel concepts and implications for studies in allergy and asthma. Allergy.

[71] Barros R., Moreira A., Padrão P., Teixeira V.H., Carvalho P., Delgado L., Lopes C., Severo M., Moreira P. (2015). Dietary patterns and asthma prevalence, incidence and control. Clin. Exp. Allergy.

[72] Tapsell L.C., Neale E.P., Satija A., Hu F.B. (2016). Foods, Nutrients, and Dietary Patterns: Interconnections and Implications for Dietary Guidelines. Adv. Nutr..

